# 

**Published:** 1860-08

**Authors:** 


### CONTENTS FOR AUGUST, 1860.

### ORIGINAL COMMUNICATIONS.

Valedictory Address of the President of the Illinois State Medical Society.............. 449
Successful Case of Ovariatomy. By WM. H. BYFORD, A. M, M. D.................. 459
Use of Ox Gall in Disease. By B. WOODWARD, M. D, Galesburg, Ill.................. 470
Stranguary as a Sign of Pregnancy. By B. McC———, M. D., Dubuque, Iowa....... 472

### BOOK AND PAMPHLET NOTICES.

Medical Uses of Electricity. By ALFRED GARRETT, M. D........................... 474
BOZEMAN on the Application of the Button Suture to Varicose Veins.................. 477
Medical Communications of the Massachusetts Medical Society.......................... 478
Coxalgia or Hip-Disease........................................................ 479

### SELECTIONS.

Pathology and Therapeutics of Typhus Fever........................................ 490
Importance of the Functions of the Skin in the Pathology and Treatment of Tubercular Consumption........................................................ 491
New York Medical and Surgical Society.—Discussion on Diphtheria.................. 493
Account of Diphtheritis, as it Occurred on the Watershed between the Tallahatchie and Mississippi Rivers........................................................ 498

### EDITORIAL.

Dipththeria---Letter from Geo. W. Slack, M. D.................................. 502
Caffeine, &c........................................................ 507
Miscellaneous Items........................................................ 511

